# Fabrication, defect chemistry and microstructure of Mn-doped UO_2_

**DOI:** 10.1038/s41598-023-50676-2

**Published:** 2024-01-18

**Authors:** H. Smith, L. T. Townsend, R. Mohun, J. F. W. Mosselmans, K. Kvashnina, Neil C. Hyatt, C. L. Corkhill

**Affiliations:** 1 Department of Materials Science and Engineering, The University of Sheffield, Sheffield, UK; 2 Diamond Light Source, Harwell Science and Innovation Campus, Didcot, UK; 3 Institute of Resource Ecology, Helmholtz-Zentrum Dresden-Rossendorf (HZDR), P.O. Box 510119, 01314 Dresden, Germany; 4 The Rossendorf Beamline at ESRF, The European Synchrotron, CS40220, 38043 Grenoble Cedex 9, France; 5 School of Mechanical and Materials Engineering, Washington State University, Pullman, WA 99164 USA; 6 School of Earth Science, The University of Bristol, Bristol, UK

**Keywords:** Energy, Nuclear fuel

## Abstract

Mn-doped UO_2_ is under consideration for use as an accident tolerant nuclear fuel. We detail the synthesis of Mn-doped UO_2_ prepared via a wet co-precipitation method, which was refined to improve the yield of incorporated Mn. To verify the Mn-doped UO_2_ defect chemistry, X-ray absorption spectroscopy at the Mn K-edge was performed, in addition to X-ray diffraction, Raman spectroscopy and high-energy resolved fluorescence detection X-ray absorption near edge spectroscopy at the U M_4_-edge. It was established that Mn^2+^ directly substitutes for U^4+^ in the UO_2_ lattice, accompanied by oxygen vacancy (O_v_) charge compensation. In contrast to other divalent-element doped UO_2_ materials, compelling evidence for U^5+^ in a charge compensating role was not found. This work furthers understanding of the structure and crystal chemistry of Mn-doped UO_2,_ which could show potential advantages as a novel efficient advanced nuclear fuel.

In so-called advanced nuclear fuels, it has been shown that by adding small amounts of Cr-dopant to the UO_2_ matrix the grain size is increased, resulting in a longer pathway for fission gasses to migrate and escape the fuel matrix, thus reducing pellet swelling during reactor operations. Other favourable properties are promoted upon Cr doping, including improved plasticity and reduced pellet-cladding interactions, all of which reduce the risk of fuel failure^1–5^. The introduction of Mn as a dopant in UO_2_ fuels has been hypothesised to offer an improved alternative to commercially available Cr-doped UO_2_ fuel^6^, warranting further study of the crystal chemistry of Mn-doped UO_2_.

Atomistic modelling of the Mn-doped UO_2_ crystal lattice and defect formation during heat treatment suggested that grain growth is enhanced by the formation of U vacancy defects (U_v_) that aid diffusion during the sintering process^7^. These defects are predicted to increase in concentration with the presence of dopants when compared to undoped UO_2_. Through atomistic modelling, the U_v_ concentration was shown to be up to five times greater in Mn-doped UO_2_ than Cr-doped UO_2_, strongly suggesting that Mn-doped UO_2_ should exhibit larger grains and, therefore, enhanced in-reactor properties with regards to fission gas migration. Experimental investigation of the synthesis of Mn-doped UO_2_ via a sol–gel method found grain size enhancement of up to 80 μm in samples when compared to UO_2_, with a measured Mn content of 490 ppm. In comparison, grains of ~ 50 μm were achieved in 350 ppm Cr-doped UO_2_ when both were sintered at 1700 °C for 6 h under a reducing Ar-4%H_2_ atmosphere^8^.

Grain size growth has also been postulated to be the result of the formation of a liquid phase in Mn-doped UO_2_, where MnO powder was dry mixed with an additional dopant, Al_2_O_3_, to form a 1000 ppm Mn/Al-doped UO_2_^9^. When sintered at 1860 °C, which is close to the melting temperature of MnO (1945 °C), a maximum grain size of ~ 50 μm was achieved, attributed to a MnO-Al_2_O_3_ binary liquid phase. The mechanism of grain growth in Mn-doped UO_2_ is, therefore, unclear and further investigation into dopant concentration and solubility of Mn in the UO_2_ lattice is warranted to optimise fuel fabrication routes.

Additionally, post-operation of a nuclear reactor, the safe storage and disposal of spent fuel is dependent on the long-term durability of the UO_2_ matrix, most prominently when exposed to geological disposal conditions^10,11^. Matrix oxidation converts the relatively insoluble U^(4+)^O_2_ to soluble U^(6+)^O_2_^2+^ and, as such, under geological disposal conditions, may result in radionuclide release into groundwater. Therefore, any alteration to UO_2_ that may result in oxidation will undoubtedly influence dissolution behaviour and so understanding how dopants affect these properties is crucial to ensuring safe disposal of radioactive waste. For example, it was recently found that the addition of Cr to UO_2_, reduced the dissolution rate of U, through a galvanic coupling effect between Cr^2+^ and U^6+^ ^12^. A thorough understanding of the crystal chemistry of Mn-doped UO_2_ in comparison to undoped UO_2_, including an investigation of defect formation, is thus required to fully underpin predictions of spent fuel dissolution behaviour over the geological disposal period (100,000 + years).

While dry-synthesis and sol–gel methods have been demonstrated for the synthesis of Mn-doped UO_2_, this work improves upon these processes using an alternative wet chemical co-precipitation synthesis route. To aid a detailed study of the local structure of Mn in UO_2_, the samples were sintered in a consistent reducing environment. Analysis of Mn-doped UO_2_, prior to, and after sintering, was performed by X-ray absorption spectroscopy (XAS), X-ray diffraction (XRD), and Raman spectroscopy. The effects of sintering in a reducing environment on the final microstructure and crystal chemistry is also discussed, completing an in-depth experimental study of Mn-doped UO_2_ crystal chemistry and providing a key foundation for future investigations of the use of Mn-doped UO_2_ as an advanced nuclear fuel.

## Results & discussion

### Development of Mn-doped UO_2_ synthesis and fabrication method

**Figure 1:**
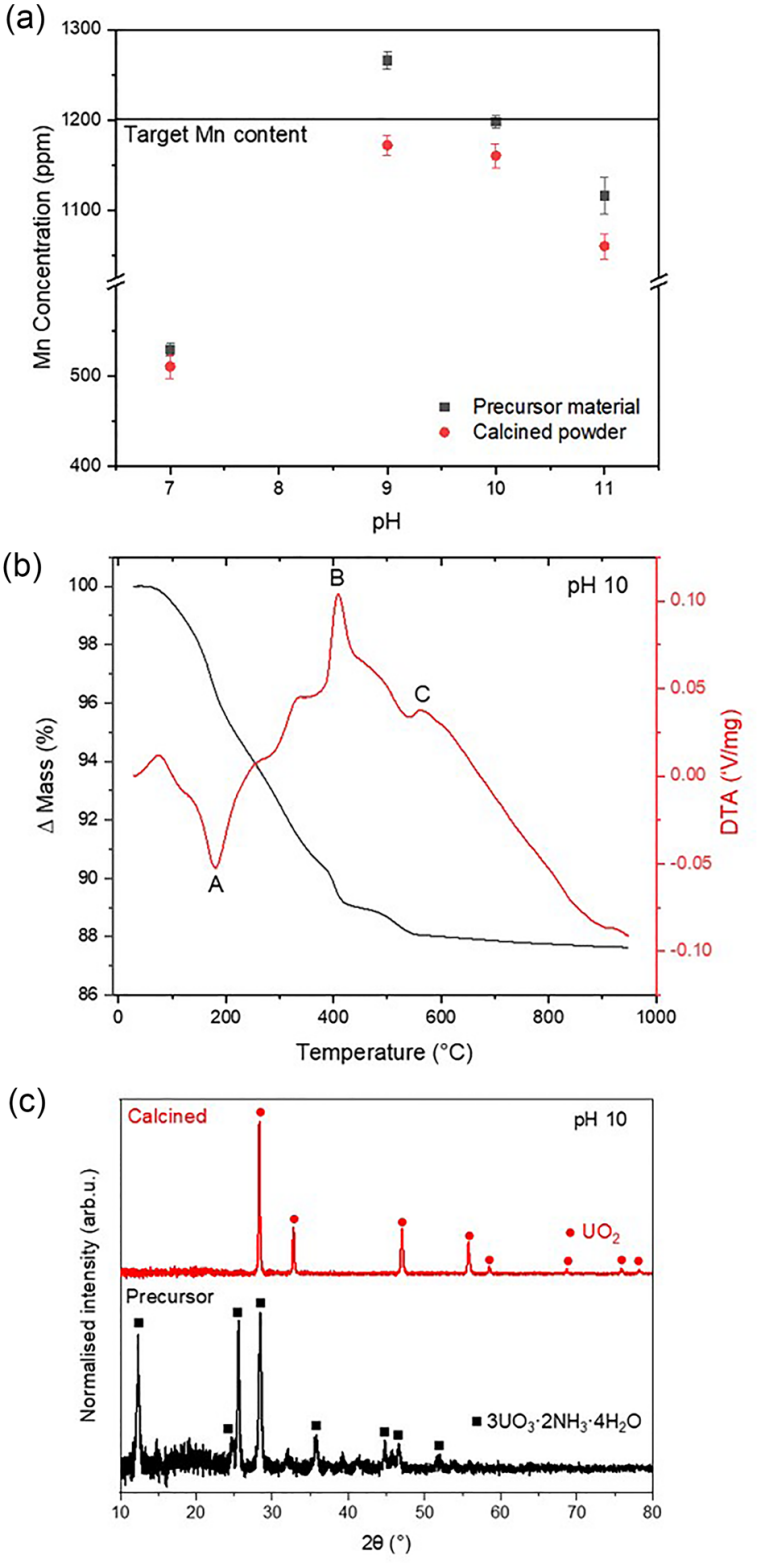
Initial investigation of an optimal synthesis and fabrication route for Mn-doped UO_2_. (**a**) Complete digest of target 1200 ppm Mn-doped UO_2_ synthesised at pH 7, 9, 10 and 11; (**b**) Thermogravimetric analysis of precursor material synthesised at pH 10 and; (**c**) X-ray diffraction patterns of precursor and calcined material (pH 10).

A nitrate co-precipitation method was developed to prepare Mn-doped UO_2_ with an initial target concentration of 1200 ppm Mn, in which nitrates of Mn (Mn(NO_3_)_3_·4H_2_O) and U (UO_2_(NO_3_)_2_·6H_2_O) were used. Concentrated NH_4_OH was added to this mixture in 0.1 mL instalments until a pH of 7, 9, 10 or 11 was reached and precipitation of a mixed compound was achieved. Analysis of the supernatants via Inductively Coupled Plasma-Optical Emission Spectroscopy indicated the success of co-precipitation of Mn for each pH, where ~ 99.00 ± 1.00% U was precipitated for all pH values. The Mn incorporation in the resulting solid upon precipitation varied, with 84.98 ± 0.01%, 99.96 ± 0.03%, 99.80 ± 0.03% and 99.9 ± 0.6% Mn incorporated for pH 7, 9, 10 and 11, respectively (Fig. 1a). This indicates that a pH of ≥ 9 is required to fully incorporate Mn within co-precipitated product. Consideration of both the supernatant analysis and a complete nitric acid digest of the solids confirmed that the optimal synthesis was achieved at pH 10 and, as such, the remainder of the samples discussed herein were fabricated using material precipitated at pH 10.

An optimal calcination temperature was determined by Thermogravimetric analysis of the precipitated precursor material (Fig. 1b), where mass losses due to the removal of water (100–190 °C)^13^, U/Mn and ammonia nitrate from synthesis (200–300 °C)^14,15^, and the decomposition of nitrate oxides formed during heating (300–525 °C)^15^, occurred up to a temperature of ~ 600 °C. The DTA curve shows endothermic peaks, A and C, relating to water loss and decomposition of nitrate oxides, respectively, and an exothermic peak, B, attributed to the formation nitrate oxides during heating. A calcination temperature of 750 °C was selected and conversion from precursor material (identified as 3UO_3_·2NH_3_·4H_2_O) to UO_2_ after heat treatment for 4 h under a reducing atmosphere (95% N_2_/5% H_2_) was confirmed via XRD of the calcined powder (Fig. 1c).

### Crystal chemistry of Mn-doped UO_2_

**Figure 2:**
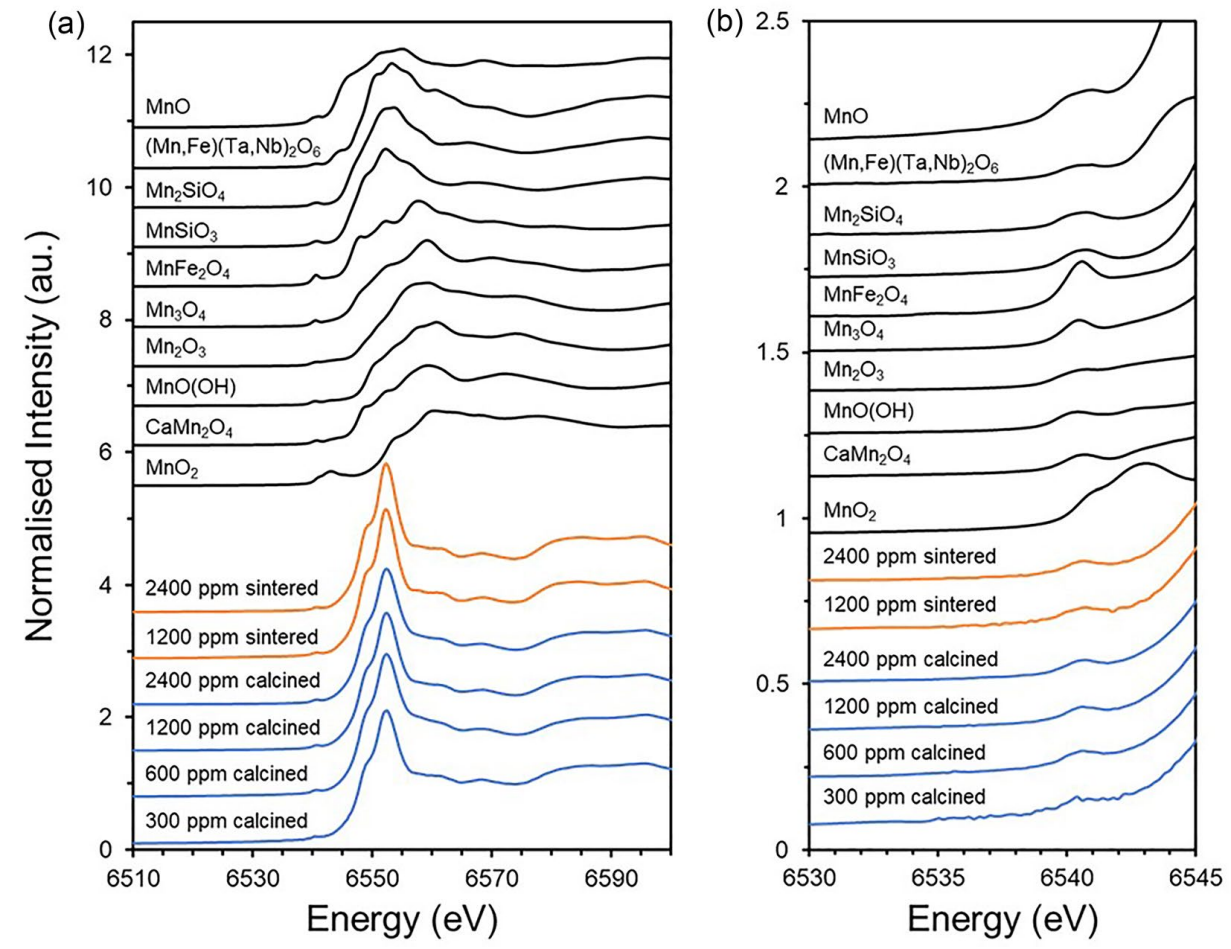
Mn K-edge XANES spectra of Mn doped UO_2_ compared to known standards. (**a**) complete spectra and; (**b**) pre-edge region. Standards are depicted in black, with calcined samples in blue and sintered samples in orange.

Mn K-edge X-ray absorption near edge spectroscopy (XANES) analysis of Mn-doped UO_2_ showed a unique coordination environment when compared to standards of known speciation (Fig. 2a). Of the different Mn standards measured, the E_0_ position (white line, taken from the peak of the first derivative) of Mn-doped UO_2_ (6551 ± 0.3 eV) was closest to that of the Mn^2+^ standards (6550–6552 ± 0.6 eV) (Table 1); however, spectrally, each Mn^2+^ standard was clearly different to the samples, suggesting that Mn in UO_2_ does not possess a local environment similar to those of the Mn standards.

**Table 1:** XANES analysis for Mn-doped UO_2_ samples and Mn standards.

Sample/Standard	Valence state	Mn environment	E_0_ (white line) position (eV, ± 0.6)	Pre-edge centroid position (eV, ± 0.6)	Pre-edge integrated area (± 0.03)
300 ppm Mn (Calcined)			6551	6540	0.049
600 ppm Mn (Calcined)			6551	6540	0.099
1200 ppm Mn (Calcined)			6551	6540	0.072
1200 ppm Mn (Sintered)	1.80^a^	Distorted cubic^b^	6551	6540	0.078
2400 ppm Mn (Calcined)			6551	6540	0.064
2400 ppm Mn (Sintered)	1.88^a^	Distorted cubic^b^	6551	6540	0.072
(Mn,Fe)(Ta,Nb)_2_O_6_	2	O_h_	6552	6540	0.055
Mn_2_SiO_4_	2	O_h_	6550	6540	0.155
MnSiO_3_	2	O_h_^c^	6551	6540	0.186
MnO	2	O_h_	6550	6540	0.286
MnFe_2_O_4_	2	T_d_	6556	6541	0.481
Mn_3_O_4_	2/3	O_h_ /T_d_	6557	6541	0.112
Mn_2_O_3_	3	O_h_	6554	6541	0.231
MnO(OH)	3	O_h_	6556	6541	0.148
CaMn_2_O_4_	3	O_h_	6555	6541	0.010
MnO_2_	4	O_h_	6558	6542	0.510

(O_h_) is octahedral and (T_d_) is tetrahedral local symmetry environment. ^a^Calculated bond valence sum from EXAFS model (Table 2); ^b^Determined from EXAFS models (Table 2); ^c^ distorted^16^.

Analysis of the pre-edge feature in the Mn K-edge XANES (Fig. 2b and Supplementary Material Fig. 1) can be used to probe the local symmetry environment^16,17^. This pre-edge feature arises from the forbidden *1s* to *3d* electronic transition and becomes more intense when there is stronger mixing of the *d* and *p* orbitals resulting from a lack of local symmetry in the Mn environment^18^. Here, the centroid position and integrated area of the pre-edge feature were similar for all Mn-doped UO_2_ samples (6540.42–6540.64 eV and 0.0490–0.0994, respectively) (Fig. 2b and Table 1). These values are in accordance with those determined for standards that contain Mn^2+^ in an octahedral local symmetry environment (6540.36–6540.83 eV and 0.055–0.285 for the centroid position and integrated area, respectively). The values for Mn-doped UO_2_ were also in agreement with literature values for (Mn^2+^,Fe)_3_Al_2_Si_3_O_12_ (6540.66 eV, 0.0745)^17^, which exhibits a cubic Mn local environment; however, the same study reports similar values for octahedrally-coordinated Mn^2+^O (6540.62 eV, 0.0745). This highlights that differentiation in the local symmetry of Mn^2+^ by pre-edge analysis is not simple and that additional Extended X-ray Absorption Fine Structure (EXAFS) analysis is required to confirm the coordination environment.

**Figure 3:**
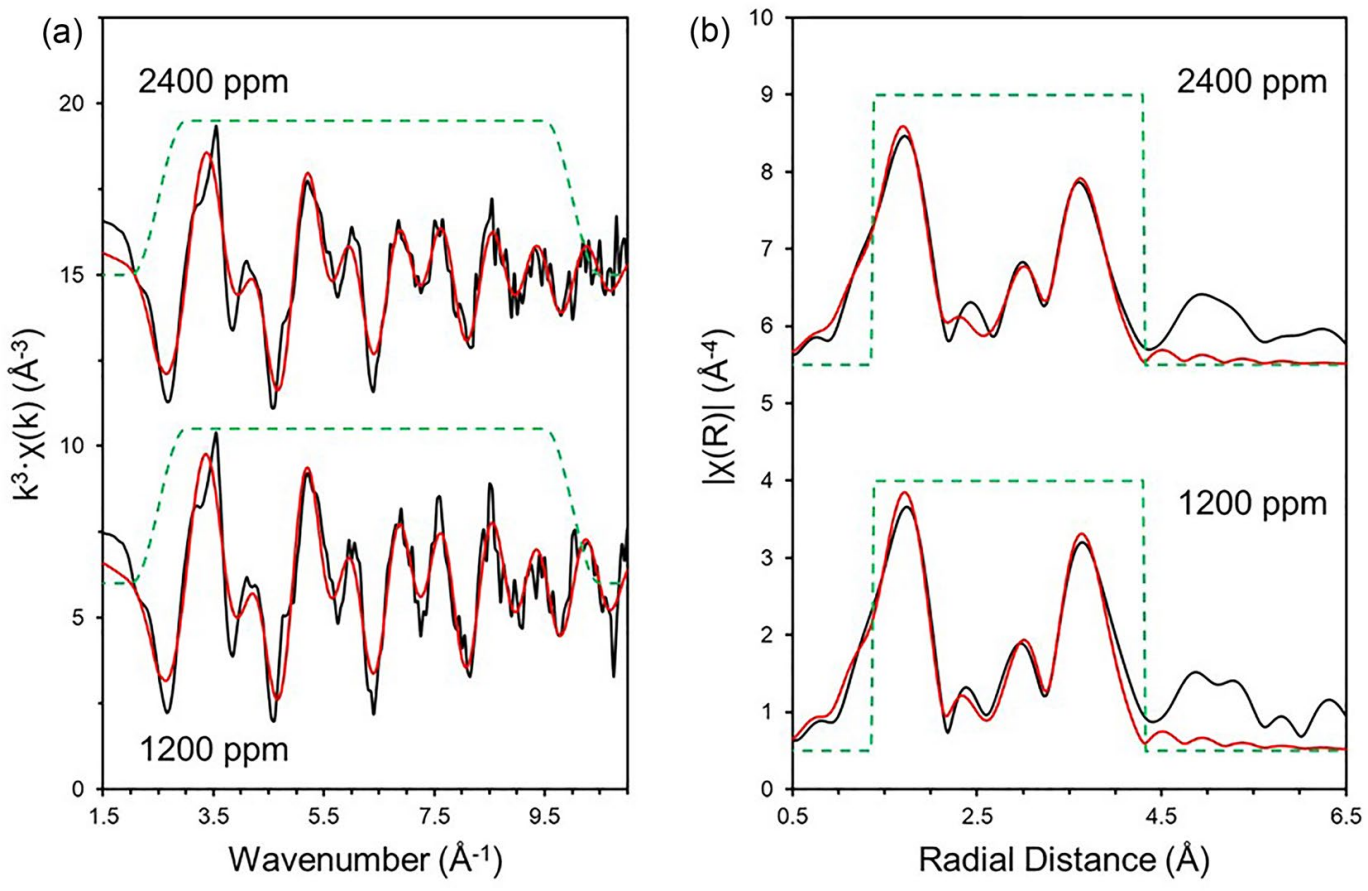
Mn K-edge spectra (black lines) and EXAFS model fits (red lines) for nominally doped 1200 ppm and 2400 ppm Mn calcined powder samples. (**a**) *k*^3^-weighted EXAFS; and (**b**) Fourier transform of the *k*^3^-weighted EXAFS, using a Hanning window function.

Since there were no changes in the XANES spectral features with increasing Mn content, nor between calcined oxide powder and sintered samples (Fig. 2), the 1200 ppm and 2400 ppm Mn-doped UO_2_ calcined powder samples were selected as representative of the local structure of Mn in UO_2_ for EXAFS fitting of the Mn K-edge. In agreement with the models of Mn-doping in UO_2_ developed by Cooper et al.^7^, the fitted data for both concentrations (Fig. 3, Table 2; corresponding data for the sintered material is given in Supplementary Material Fig. 2), indicated that the first O nearest neighbour environment of Mn is split between 6 O atoms at 2.27–2.29 ± 0.01 Å and 2 O atoms at 2.55–2.57 ± 0.03 Å in an eightfold coordination environment, consistent with direct substitution of Mn onto the U^4+^ site, or onto an interstitial site^7^. Slight contraction in the first Mn–O distance, when compared to 2.36 ± 0.02 Å U–O distance, is to be expected if Mn is substituted onto the U^4+^ site, due to the smaller cation size of Mn^2+^ (0.96 Å) compared to U^4+^ (1.00 Å). This contraction also creates a small amount of distortion in the local Mn environment, which is likely the reason for the first coordination shell splitting into two different O distances.

**Table 2:** Fitting parameters and results for 1200 ppm and 2400 ppm Mn-doped UO_2_ oxide powders.

	UO_2_ + 1200 ppm Mn (Calcined oxide powder)	UO_2_ + 2400 ppm Mn (Calcined oxide powder)
**S**_**0**_^2^	1.0	1.0
∆E_o_	− 0.8 (1)	− 0.9 (6)
N (Mn-O1)	6	6
R_eff_ (Mn-O1)	2.37	2.37
R(Mn-O1)	2.29 (1)	2.27 (1)
σ^2^ (O1)	0.016 (1)	0.018 (1)
α (%)	100.0	100.0
N (Mn-O2)	2	2
R_eff_ (Mn-O2)	2.37	2.37
R(Mn-O2)	2.57 (3)	2.55 (3)
σ^2^ (O2)	0.012 (4)	0.015 (4)
α (%)	100.0	100.0
N (Mn-U1)	5	5
R_eff_ (Mn-U1)	3.87	3.87
R(Mn-U1)	3.78 (1)	3.78 (1)
σ^2^ (U1)	0.011 (1)	0.013 (1)
α (%)	100.0	100.0
R-factor	0.0098	0.0071
Bond valence sum (O1)	1.56	1.62
Bond valence sum (O2)	0.24	0.26
Bond valence sum (total)	1.80	1.88

(S_0_^2^) is the amplitude reduction factor, (∆E_0_) the shift from Mn K-edge position (5.989 keV), (N) the degeneracy, (R_eff_) (Å) the reference bond length, (R) (Å) the fitted bond length, (σ^2^) the Debye–Waller factor and (α) F-test factor. EXAFS data and fits are shown in Fig. 4

Substitution of Mn^2+^ on the U^4+^ lattice site is further supported by a decrease in the lattice parameter with increasing Mn content, consistent with Vegard’s Law (Fig. 4). Both calcined powder and sintered samples maintained the fluorite crystal structure of UO_2_ (Supplementary Material Fig. 3)_._ Beyond the first coordination shell, a further 5 U atoms were fit at distance of 3.78 ± 0.01 Å (Table 2). The decreased coordination from the expected 12 U atoms at 3.85 ± 0.06 Å in crystalline UO_2_ may be attributed to a combination of lattice distortion from Mn-doping and of the limited data range available (2–10.5 Å^−1^). The combination of these two factors makes delineation of the distorted U-U backscatterers challenging; extension of the data range, not practically possible in this experiment due to the low Mn concentrations within a highly absorbing UO_2_ matrix, could resolve this.

**Figure 4:**
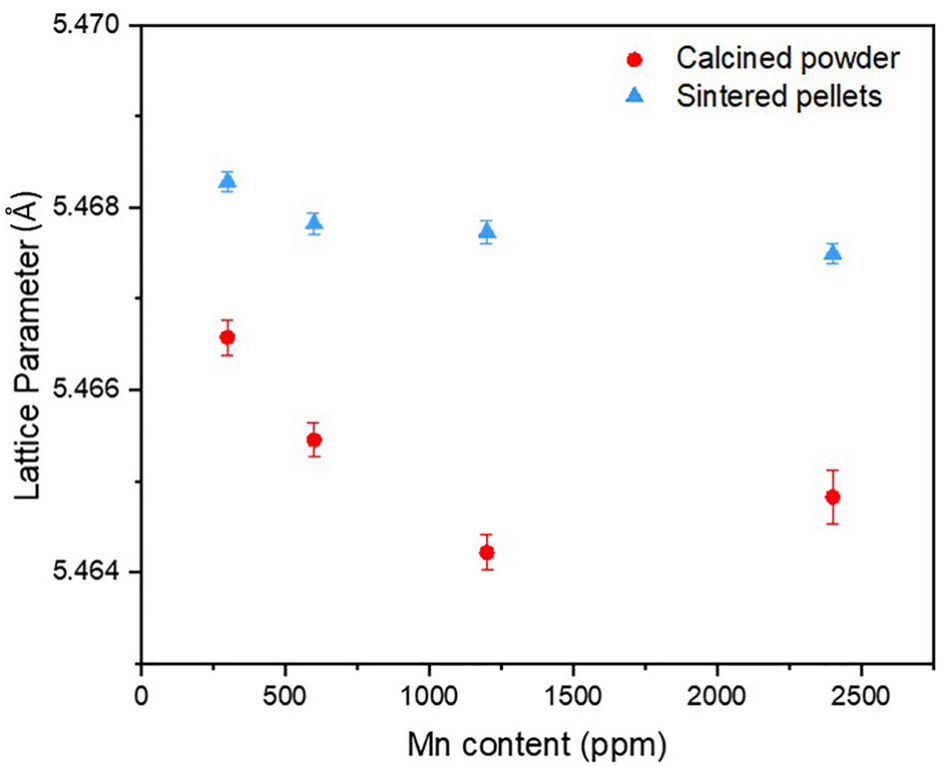
Lattice parameter values for calcined powder and sintered material, as determined by XRD. Errors are the standard deviation of triplicate analysis.

The bond valence sum (BVS) was calculated and used to indicate the oxidation state. Values of 1.80 and 1.88 were calculated for the 1200 ppm and 2400 ppm Mn-doped UO_2_ samples, respectively, supporting the presence of Mn^2+^ (Table 2). This is in agreement with recently published studies of the coordination of Cr doped in the UO_2_ structure, which was found as Cr^2+^ in calcined material^19^.

**Figure 5:**
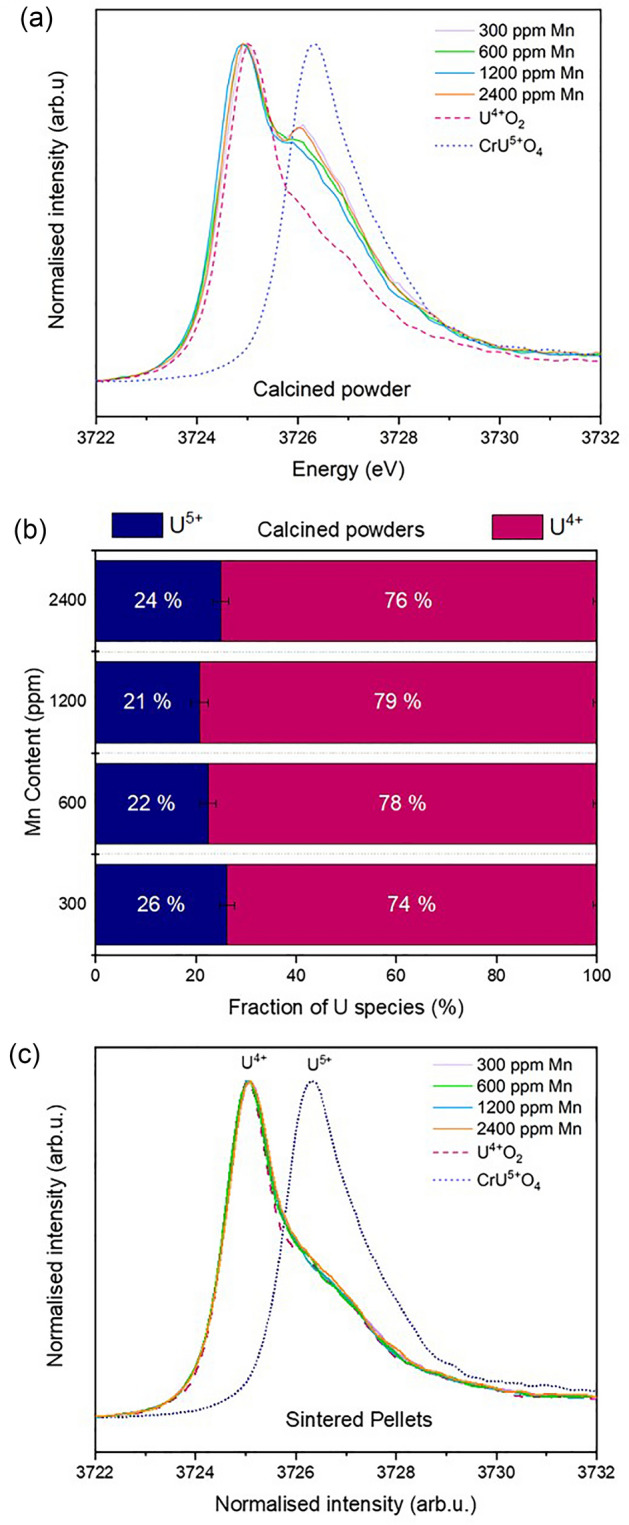
U M_4_-edge HERFD XANES of Mn-doped UO_2_ materials. (**a**) calcined material; (**b**) quantification of the U^4+/5+^ ratio in calcined powder determined from fitting of U M_4_-edge HERFD XANES data in (**a**); and (**c**) sintered material.

A charge balance mechanism is required in the substitution of Mn^2+^ on the U^4+^ site, provided by the formation of positive defects, U^5+^ and/or oxygen vacancies (O_v_). Experimental evidence for U^5+^ formation can be determined using the High Energy Resolution Fluorescence Detected (HERFD) XANES method, with data acquired at the U M_4_-edge; however, given that the doping level of Mn was low (300–2400 ppm), the amount of corresponding U^5+^ (up to 0.24%) is at, or below, the limits of detection of the technique. Nevertheless, principal component analysis of the data series revealed that, for the calcined powder, two components, attributed to U^5+^ (closely matching a standard of CrUO_4_) and U^4+^ (UO_2_) oxidation states, were required to accurately reconstruct each of the samples in the series (Fig. 5a and Supplementary Material Fig. 4).

Iterative target transformation factor analysis was used to indicate the approximate fraction of U^4+^ and U^5+^ in each of the oxide powders (Fig. 5b), with results indicating ~ 21–26 ± 2% U^5+^ present in Mn-doped UO_2_ (and, therefore, ~ 74–79 ± 1% U^4+^). Given the arguments above, the presence of U^5+^ seems unlikely to arise from a charge compensation mechanism, but from oxidation of the samples. Despite being exposed to air post-synthesis for the same period of time as the calcined samples, the sintered materials did not show any strong evidence for the presence of U^5+^ (Fig. 5c).

**Figure 6:**
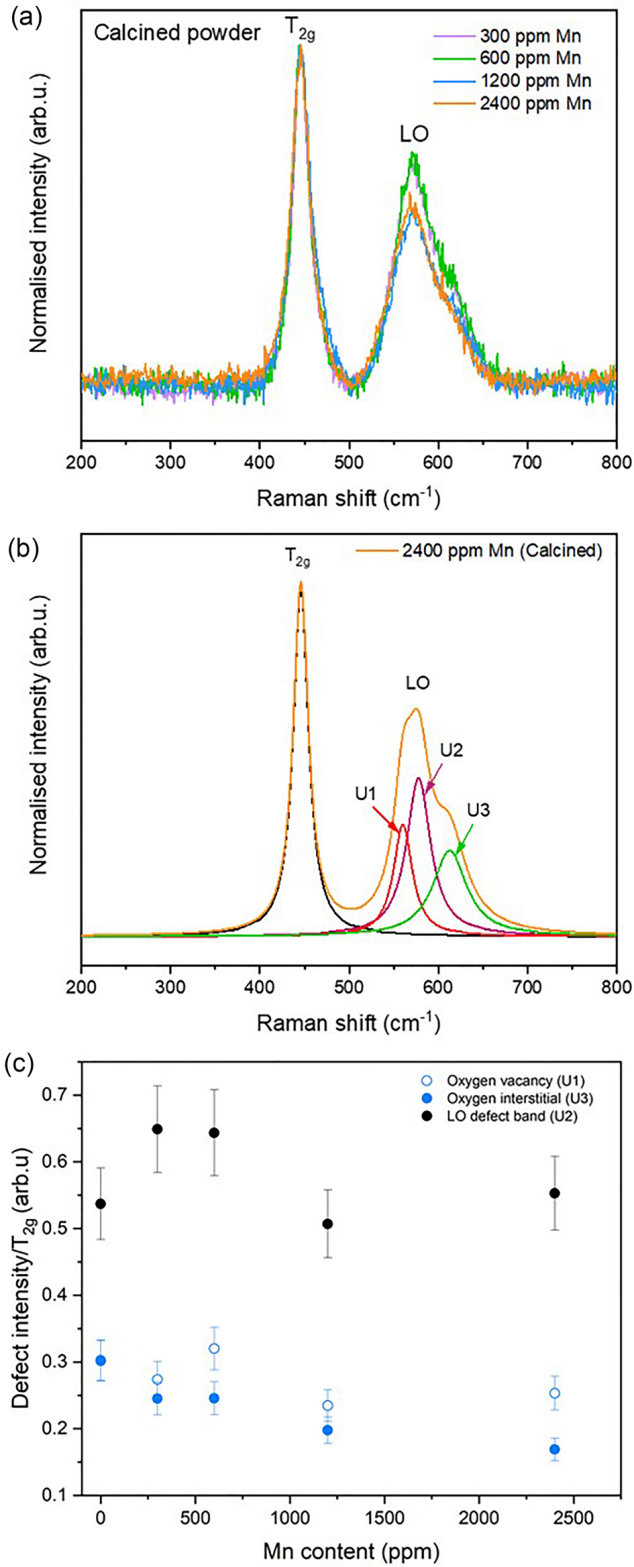
Raman analysis of Mn-doped UO_2_ calcined powders. (**a**) Raman spectra; (**b**) deconvolution of the 2400 ppm Mn-doped UO_2_ spectra into U1, U2 and U3 peaks; and (**c**) defect content realised by the area ratio of the defect peak to the T_2g_ peak.

The formation of O_v_ defects was observed through Raman spectroscopy (Fig. 6a). The main T_2g_ peak (~ 445 cm^−1^) of UO_2_ and the defect band (~ 500–700 cm^−1^), indicative of lattice distortion due to defect formation, was present for all samples. The relative intensity of individual peak contributions of the defect bands, where U1 is attributed to O_v_, U2 to the LO phonon mode and U3 to O_i_, was compared to the relative intensity of the T_2g_ peak^20^ (Fig. 6b). There were minor fluctuations in the relative contributions as the concentration of Mn was increased; however, O_v_ were always found to be present (Fig. 6c), confirming their formation as a charge balance mechanism for Mn doping in UO_2_.

As noted previously, there were no significant differences in the crystal structure of sintered Mn-UO_2_ when compared to the calcined oxide powders, indicated by consistency in spectral features observed in the Mn K-edge XANES (Fig. 2) and the Fourier Transform of the EXAFS region (Fig. 3 and Supplementary Material Fig. 2). The reduction in UO_2_ lattice parameter, due to Mn^2+^ substitution on the U^4+^ site, was maintained; although, the absolute values of lattice parameter were 0.002–0.004 Å greater in the sintered samples than the calcined samples (Fig. 4). This could be the result of volatilisation of Mn during sintering (see below) and/or reduction of U^5+^ (0.84 Å) to U^4+^ (1.00 Å) imposed by the reducing high temperature environment, as observed by HERFD-XANES (Fig. 5c), in which no U^5+^ was present.

### Influence of reducing sintering conditions on the microstructure of Mn-doped UO_2_.

**Figure 7:**
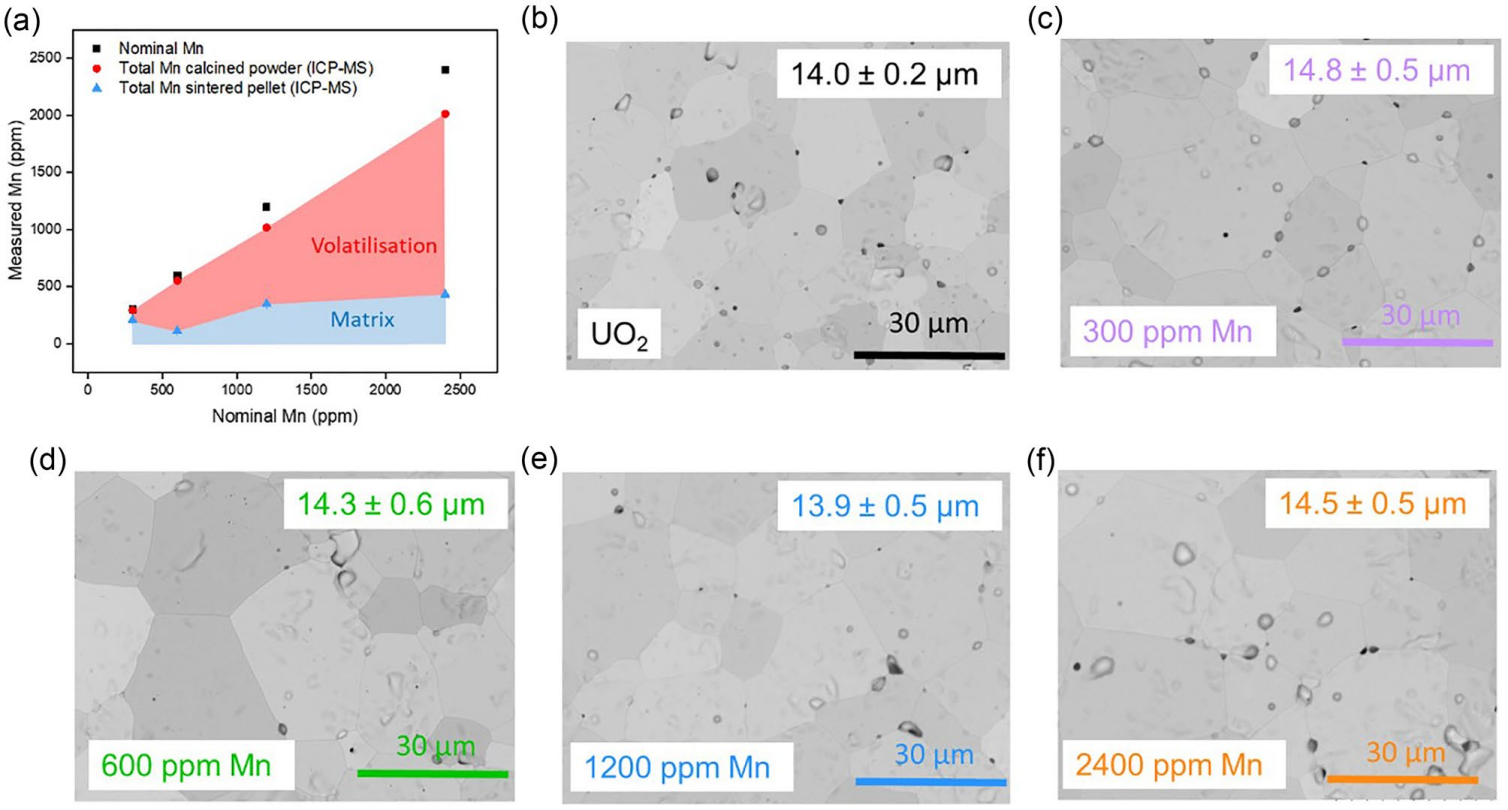
Microstructural evolution of undoped and Mn-doped UO_2_ sintered in a reducing environment. (**a**) the measured Mn content in Mn-doped UO_2_ calcined powder and sintered pellets; and SEM images and average grain size of ~ 500 grains measured across the sample surface in (**b**) undoped UO_2_ and (**c**–**f**) Mn-doped UO_2_ of increasing Mn content.

When comparing the Mn concentrations of calcined oxide powders with those of materials sintered in a reducing (95% N_2(g)_ / 5% H_2(g)_) environment, it is evident that significant volatilisation of Mn occurred (Fig. 7a)^8^. For UO_2_ doped with 300 ppm Mn, the loss was ~ 30%, while for the remaining concentrations (600, 1200 and 2400 ppm), it was ~ 80%.

No significant influence on the grain size was observed when the average measurements of ~ 500 grains were considered in comparison to undoped UO_2_ (Fig. 7b–e); however, there was a marginal increase with increasing dopant concentration. An average grain size of 13.9–14.8 ± 0.5 μm was recorded, in comparison with undoped UO_2_, sintered under the same conditions, which had an average grain size of 14.0 ± 0.2 μm. This is in contrast to previous studies^8^ and modelling predictions^6^, most likely due to the nature of the sintering atmosphere, which contained no O_2_.

Studies of commercially available Cr-doped UO_2_ have shown that the sintering atmosphere is the most important factor contributing to grain growth, with the addition of small quantities of oxygen thought to promote the formation of a ‘CrO’ eutectic phase. At sintering temperatures (> 1675 °C), this phase is liquid and aids grain growth, evidenced as precipitates of Cr_2_O_3_ formed upon cooling^21–24^. The effects of a eutectic phase have been reported for Mn/Al doped UO_2_ sintered in a H_2_ atmosphere at 1860 °C, where a MnO/Al_2_O_3_ phase was observed by EDX mapping^10^. However, contrary to Cr-doped UO_2_, the addition of oxygen to the sintering atmosphere in Mn/Al doped UO_2_ resulted in a smaller grainsize, attributed to increased solubility of MnO in the UO_2_ matrix^10^.

In the present study, under fully reducing conditions, much of the Mn^2+^ incorporated into the UO_2_ was volatilised, and no precipitates were observed, which indicates that the solubility limit of Mn in UO_2_ was not reached and that an equivalent MnO liquid phase was not likely formed. Moreover, no significant increase in the grain growth was observed with increasing Mn concentration (Fig. 7). One reason for this is that the temperature of sintering (1700 °C) was below the melting point of MnO_2_ (1945 °C), therefore, the formation of any possible eutectic phase was restricted. It has been previously shown, in Cr-doped UO_2_, that, if the sintering atmosphere required for the eutectic phase formation is not met, the presence of precipitates at grain boundaries will have the opposite effect and grain growth will be hindered by grain boundary “pinning”^21^; however, this reasoning is excluded here due to the absence of precipitates in the Mn-doped UO_2_ samples.

Despite the observation that the average grain size did not significantly increase upon increased levels of Mn doping, when compared with the undoped UO_2_, individual grain sizes of up to 50 μm were observed for all Mn-doped UO_2_ samples studied (Fig. 7b–e). This ‘localised’ grain growth has previously been reported for Mn-doped UO_2_ prepared via sol–gel synthesis and sintered under reducing conditions (95% Ar_(g)_ / 5% H_2(g),_ 6h, 1700 °C)^8^. Such behaviour may be due to the increased diffusivity of U atoms predicted by atomistic models of the defect concentrations^7^. Further work to underpin the solubility limit of Mn in UO_2_, and to develop an understanding of the influence of sintering conditions on Mn–UO_2_, is required to fully elucidate the mechanism of grain size growth.

These results have given insight into the crystallographic basis for interpretation of grain growth as well as oxidation and dissolution behaviours in Mn-doped UO_2_ and support development for future use as accident tolerant fuel.

## Methods

### Sample preparation

**Figure 8:**
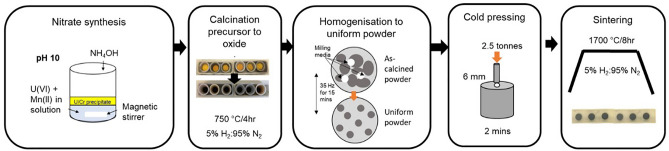
Optimised synthesis and fabrication route for Mn- doped UO_2_.

A wet synthesis method was developed using uranium nitrate hexahydrate (UO_2_(NO_3_)_2_·6H_2_O, (The British Drug House (BDH). B.D.H Laboratory Chemicals Division, > 98%, 0.3 mol L^−1^). This was mixed with manganese(II) nitrate tetrahydrate (Mn(NO_3_)_3_·4H_2_O, Sigma Aldrich, 99.99%, 1.6 mol L^−1^) in the proportion required to give the desired concentration of dopant. An initial synthesis study was performed in which a number of samples were assessed to determine the optimal pH and calcination temperature for fabrication of Mn-doped UO_2_. The final fabrication route is shown in Fig. 8 and is detailed as follows. Concentrated ammonium hydroxide, NH_4_OH (5 mol L^−1^), was added until a pH of 10 was reached. The resultant precipitate was vacuum filtered, washed in ultra-high quality (18 MΩ cm) water, and dried for 24 h at 90 °C. Thermogravimetric analysis was carried out on precursor material using a Netzsch TG 449 F3 Jupiter instrument coupled with a 64-channel QMS 403 D Aeolos mass spectrometer. Samples were heated to 1000 °C at a rate of 10 °C min^−1^ under a constant Ar_(g)_ flow. Analysis of the mass loss over time determined that a calcination temperature of 750 °C for 4 h under a reducing (95% N_2(g)_ / 5% H_2(g)_) atmosphere was sufficient to convert the precursor material to oxide. Successful co-precipitation of Mn with U oxide was measured by inductively coupled plasma optical emission spectroscopy (ICP-OES) analysis of the supernatant, where > 99.9% precipitation was observed for both U and Mn for samples precipitated at pH 10.

Homogenised oxide powders, prepared via milling at 35 Hz for 15 min, were uniaxially pressed at 2.5 tonnes into 6 mm green pellets and sintered at 1700 °C for 8 h in a reducing atmosphere (95% N_2(g)_ / 5% H_2(g)_). Complete digest of precursor and calcined material, as well as sintered pellets (crushed to powder using a mortar and pestle), was carried out to assess the Mn content. In this method, 20 mg of sample was completely dissolved in 2M nitric acid (ultrapure HNO_3_) at 90 °C, with the aid of a magnetic stirrer, and an aliquot was removed and analysed by ICP-OES (Thermofisher Scientific iCAPDuo6300) for Mn and U concentration. Samples were dissolved in triplicate and an average measurement of Mn, with errors of 1 standard deviation, for each sample reported.

### Characterisation

Secondary electron images of thermally etched pellet sample surfaces were taken using a Hitachi TM3030 scanning electron microscope at an accelerating voltage of 15 kV. Samples were thermally etched at 80–90% sintering temperature under a reducing atmosphere (95% N_2(g)_ / 5% H_2(g)_) and the revealed grains imaged at 500 × magnification. The Image J software was used to obtain the average grain size of ~ 500 grains in images taken from 5 different regions across the pellet surface.

A PANalytical Xpert3 diffractometer was used in reflection mode with a 45 keV/40 mA generator to characterise powder samples by XRD (XRD). Data were collected between 5° and 100° 2θ with a step size of 0.013° and a step time of 40 s, a fixed slit size of 0.5 was used. LaB_6_ (20–30 wt%) was used as an internal standard for data alignment, corrected using the WinXPow software. Accurate determination of lattice parameters was carried out in the Topas software, using Le Bail refinements. To avoid oxidation, calcined powders were measured immediately post heat treatment (i.e. within 10 min of removal from the furnace) and sintered samples were crushed upon removal from the furnace, within a controlled inert atmosphere (N_2(g)_), and immediately measured to avoid oxidation.

Mn K-edge (6.539 keV) and U M_4_-edge (3.728 keV) X-ray Absorption Spectroscopy measurements were performed, at room temperature, on both calcined powder and sintered Mn-doped UO_2_. The I20-scanning beamline at the Diamond Light Source (DLS), UK, was used in fluorescence mode to measure the Mn K-edge using a 64-element Ge detector with Xspress4 signal processing and a beam size of 400 × 300 µm (FWHM). A high flux and excellent energy resolution is given by the wiggler-sourced I20-scanning beamline due to the use of a Si(111) four-bounce crystal monochromator, allowing the detection of low concentrations of Mn dopant in the heavily absorbing UO_2_ matrix.

Multiple scans were taken across a number of energy ranges to improve data quality. Energy steps of 5 eV were taken from 6439 to 6535 eV, 0.2 eV from 6536 to 6560 eV with a time step of 1 s step^−1^ in the XANES region for all samples. In the EXAFS region for calcined Mn-doped UO_2_ samples, an energy step of 0.04 Å^−1^ was taken from 6560 – 6980 eV with a time step of 1–6 s step^−1^. The spectral features in the sintered samples were identical to those of the calcined powers. A number of standards of known Mn valence state and coordination environment were measured in transmission mode, including a range of Mn^2+^ standards: Mn^2+^O; (Mn^2+^, Fe)(Ta, Nb)_2_O_6_; Mn^2+^SiO_6_; and Mn^2+^_2_SiO_4_.

The E_0_ (white line, taken from the peak of the first derivative) positions of the raw data were aligned to the E_0_ position of a standardised reference Mn foil that was measured simultaneously with Mn-doped UO_2_ samples. A cubic spline background subtraction and normalisation procedure^25^ was then carried out and multiple scans merged using the Athena software^26^. To isolate the pre-edge region for analysis, an arctangent function was applied across the 6535.0–6548.0 eV energy range to remove the photoelectric background absorption^27^ and three Gaussian peak functions used to model the pre-edge features (Supplementary Material Fig. 1). The energy position and normalised height were refined using the non-linear least-square fitting SOLVER function in Excel and the pre-edge centroid position assigned by taking the intensity weight average of each Gaussian fit.

To create models of the local environment realised in the EXAFS region, absorption data (eV) were converted to wavenumber, k (Å^−1^), and a Fourier Transform of the resulting k space was used for fitting. The possible scattering paths of the Mn central absorber atom to the surrounding atoms in the Mn-doped UO_2_ local environment were generated using a FEFF6 algorithm^28^ in Artemis^26^ where a UO_2_ CIF file was modified to include Mn as the central absorbing atom at the first U lattice position. The range of k (Å^−1^), r (Å), and the amplitude reduction factor (S_0_^2^) were optimised for the data and the following parameters allowed to refine: degeneracy (N); fitted bond length (R) (Å); shift from Mn K-edge position (6.539 keV) (∆E_0_) and; the Debye–Waller factor (σ^2^). The F-test was applied to each fitted path, the result (α) indicated the confidence of adding the path to improve the fit (> 67% gives a confidence of 1σ, > 95% gives a confidence of 2σ)^29^. The bond valence sum (BVS) was calculated for the Mn–O coordination environment, which is an evaluation of bond distances in a coordination shell that are equal to the formal oxidation state of the cation absorber^30^.

HERFD XANES was carried out at the U M_4_-edge using the BM20 beamline at the European Synchrotron (ESRF), France^31^, with the use of an X-ray emission spectrometer^32^. Calcined powders were mixed with poly-ethylene glycol (PEG) and pressed into 6 mm pellets for analysis, while the as-sintered surfaces of pellets were measured. Raw U M_4_-edge data was pre-processed in Athena, as discussed above, and a principal component analysis was used to determine the possible number of spectral components, followed by Iterative Target Transformation Factor Analysis (ITTFA)^33^ to indicate the relative concentration of each component using U^(4+)^O_2_ and CrU^(5+)^O_4_ standards measured alongside Mn-doped UO_2_ at the BM20 beamline.

Raman measurements were performed using a Renishaw inVia Reflex confocal spectrometer equipped with a Leica DM2500 microscope. Calcined Mn-doped UO_2_ powders were pressed into pellets before analysis using a 514.5 nm green argon laser with 1800 lines mm^−1^ grating to acquire a spectral acquisition between 200 and 800 cm^−1^. An average of 10 points across the compacted green pellet surface of each pellet was taken, to confirm their reproducibility and homogeneity of the composition across the sample. Lorentz function fitting was used to obtain information of peaks attributed to defects (U1, U2 and U3) in the UO_2_ structure, relative to the main T_2g_ band of UO_2_ at 445 cm^−1^. In addition to the U2 LO phonon band at ~ 574 cm^−1^, the U1 peak at ~ 527 cm^−1^ attributed to O_v_ defects, and U3 peak at ~ 634 cm^−1^ attributed to O_i_ defects, were identified^19^.

### Supplementary Information

Supplementary Figures.

## Data Availability

The data that support the findings of this study are available from the corresponding author, upon reasonable request.

## References

[1] Arborelius J (2006). Advanced doped UO_2_ pellets in LWR applications. J. Nucl. Sci. Technol..

[2] Massih, A. Effects of additives on uranium dioxide fuel behaviour, *Swedish Radiat. Saf. Auth.* 74 (2014).

[3] Killeen JC (1980). Fission gas release and swelling in UO_2_ doped with Cr_2_O_3_. J. Nucl. Mater..

[4] Kashibe S, Une K (1998). Effect of additives (Cr_2_O_3_, Al_2_O_3_, SiO_2_, MgO) on diffusional release of ^133^Xe from UO_2_ fuels. J. Nucl. Mater..

[5] Une K, Tanabe I, Oguma M (1987). Effects of additives and the oxygen potential on the fission gas diffusion in UO_2_ Fuel. J. Nucl. Mater..

[6] Cooper, M. W. D., Andersson, A. D. R. & Stanek, C. R. *Mn-Doped Oxide Nuclear Fuel*, United States Patent, US 10,847,271 B1 (2020).

[7] Cooper MWD, Stanek CR, Andersson ADR (2018). The role of dopant charge state on defect chemistry and grain growth of doped UO_2_. Acta Mater..

[8] Finkeldei, S. C. *et al.**Synthesis and characterization of UO*_*2*_* feedstocks containing controlled dopants.* Oak Ridge National Laboratory Report, ORNL/SPR-2019/1067 (2019).

[9] Kang KW (2010). Effects of MnO–Al_2_O_3_ on the grain growth and high-temperature deformation strain of UO_2_ fuel pellets. J. Nucl. Sci. Technol..

[10] Ewing RC (2015). Long-term storage of spent nuclear fuel. Nat. Mater..

[11] Nuclear Decommissioning Authority. *Geological Disposal: Package Evaluation Status Report*, NDA Report no. SCCS/451/01 (2016).

[12] Smith H (2023). Oxidative dissolution of Cr-doped UO_2_ nuclear fuel. npj Mater. Degrad..

[13] Martinez J (2015). From uranium(IV) oxalate to sintered UO_2_: Consequences of the powders thermal history on the microstructure. J. Eur. Ceram. Soc..

[14] De Bruijn TJW, De Ruiter GMJ, De Jong WA, Van Den Berg PJ (1981). Thermal decomposition of aqueous manganese nitrate solutions and anhydrous manganese nitrate. Part 2. Heats of reaction. Thermochim. Acta.

[15] Rajagopalan KV, Ravindran PV, Radhakrishnan TP (1995). Thermal Decomposition of uranyl nitrate hexhydrate: A thermal analysis - mass spectrometry study. J. Therm. Anal..

[16] Farges F (2005). *Ab initio* and experimental pre-edge investigations of the Mn K -edge XANES in oxide-type materials. Phys. Rev. B.

[17] Chalmin E, Farges F, Brown GE (2009). A pre-edge analysis of Mn K-edge XANES spectra to help determine the speciation of manganese in minerals and glasses. Contrib. Mineral. Petrol..

[18] Dräger G, Kirchner T, Bocharov S, Kao CC (2001). Spin-resolved NEXAFS from resonant X-ray scattering. Int. Union Crystallogr..

[19] Smith H (2022). Cr^2+^ solid solution in UO_2_ evidenced by advanced spectroscopy. Comm. Chem..

[20] Guimbretière G (2012). Determination of in-depth damaged profile by Raman line scan in a pre-cut He^2+^ irradiated UO_2_. Appl. Phys. Lett..

[21] Bourgeois L, Dehaudt P, Lemaignan C, Hammou A (2001). Factors governing microstructure development of Cr_2_O_3_-doped UO_2_ during sintering. J. Nucl. Mater..

[22] Kuri G (2014). Local atomic structure of chromium bearing precipitates in chromia doped uranium dioxide investigated by combined micro-beam X-ray diffraction and absorption spectroscopy. J. Nucl. Mater..

[23] Leenaers A, De Tollenaere L, Delafoy C, Van den Berghe S (2003). On the solubility of chromium sesquioxide in uranium dioxide fuel. J. Nucl. Mater..

[24] Cardinaels T (2012). Chromia doped UO_2_ fuel: Investigation of the lattice parameter. J. Nucl. Mater..

[25] Newville M (2001). IFEFFIT: Interactive XAFS analysis and FEFF fitting. J. Synchrotron Radiat..

[26] Ravel B, Newville M (2005). ATHENA, ARTEMIS, HEPHAESTUS: Data analysis for X-ray absorption spectroscopy using IFEFFIT. J. Synchrotron Radiat..

[27] Waychunas GA (1987). Synchrotron radiation XANES spectroscopy of Ti in minerals: Effects of Ti bonding distances, Ti valence, and site geometry on absorption edge structure. Am. Mineral..

[28] Ankudinov A, Ravel B (1998). Real-space multiple-scattering calculation and interpretation of X-ray absorption near-edge structure. Phys. Rev. B Condens. Matter Mater. Phys..

[29] Downward L, Booth CH, Lukens WW, Bridges F (2007). A variation of the F-test for determining statistical relevance of particular parameters in EXAFS fits. AIP Conf. Proc..

[30] Brown ID, Altermatt D (1985). Bond-valence parameters obtained from a systematic analysis of the inorganic crystal structure database. Acta Crystallogr. Sect. B..

[31] Scheinost AC (2021). ROBL-II at ESRF: A synchrotron toolbox for actinide research. J. Synchrotron Radiat..

[32] Kvashnina KO, Scheinost AC (2016). A Johann-type X-ray emission spectrometer at the Rossendorf beamline beamlines. J. Synchrotron Radiat..

[33] Rossberg A (2009). Identification of uranyl surface complexes on ferrihydrite: Advanced EXAFS data analysis and CD-music modeling. Environ. Sci. Technol..

[34] Hyatt NC (2020). The HADES facility for high activity decommissioning engineering & science: Part of the UK national nuclear user facility. IOP Conf. Ser. Mater. Sci. Eng..

