# Supplementary material for: Fabrication, defect chemistry and microstructure of Mn-doped UO2
Source: Sci Rep. 2024 Jan 18;14:1656. doi: 10.1038/s41598-023-50676-2 (PMC10796358; doi:10.1038/s41598-023-50676-2)
Supplement: Supplementary file 1 — Supplementary Figures. [file 41598_2023_50676_MOESM1_ESM.docx]

**Fabrication, Defect Chemistry and Microstructure of Mn-doped UO_2­_**

H. Smith^1^, L. T. Townsend^1^, R. Mohun^1^, J. F. W. Mosselmans^2^, K. Kvashnina^3,4^, Neil C. Hyatt^5,6^, C. L. Corkhill^1,6*^

^1^ Department of Materials Science and Engineering, The University of Sheffield, UK

^2^ Diamond Light Source, Harwell Science and Innovation Campus, Didcot, UK

^3^ Helmholtz-Zentrum Dresden-Rossendorf (HZDR), Institute of Resource Ecology, PO Box 510119, 01314, Dresden

^4^ The Rossendorf Beamline at ESRF – The European Synchrotron, CS40220, 38043 Grenoble Cedex 9, France

^5^School of Mechanical and Materials Engineering, Washington State University, Pullman, WA, 99164, USA

^6^ School of Earth Science, The University of Bristol, Bristol, UK

*Corresponding Author: [c.corkhill@bristol.ac.uk](mailto:c.corkhill@bristol.ac.uk)


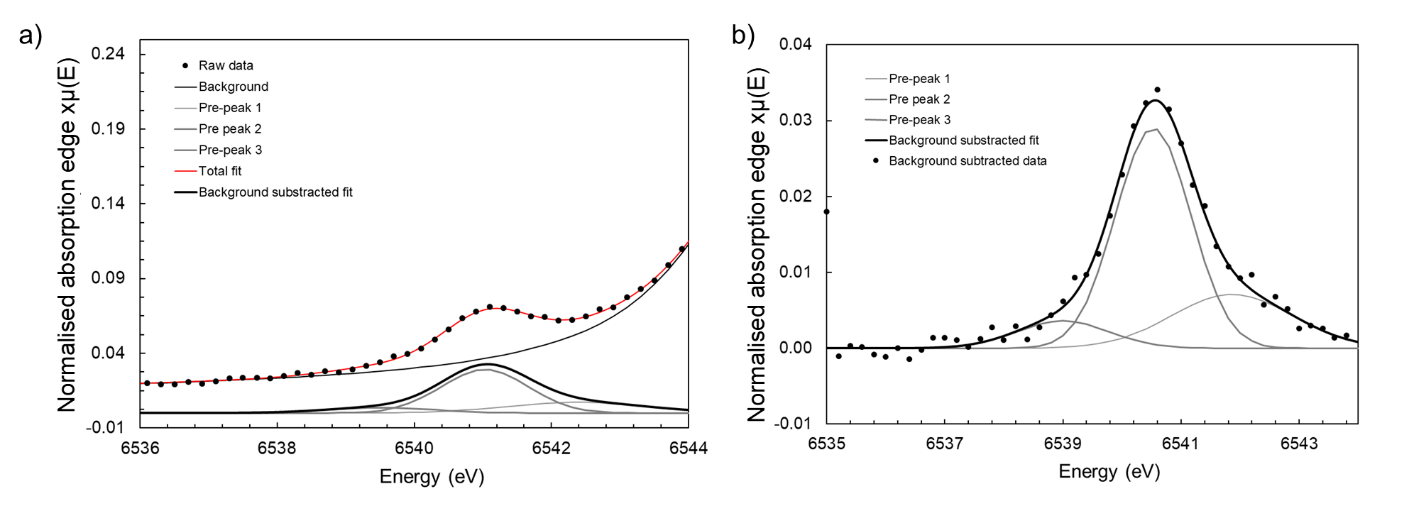


**Supplementary Figure 1. Pre-edge fitting of Mn k-edge XANES of calcined material**. **a** example pre-edge fit; and **b** Gaussian fitting of the isolated pre-edge feature of 2400 ppm Mn-doped calcined oxide powder.


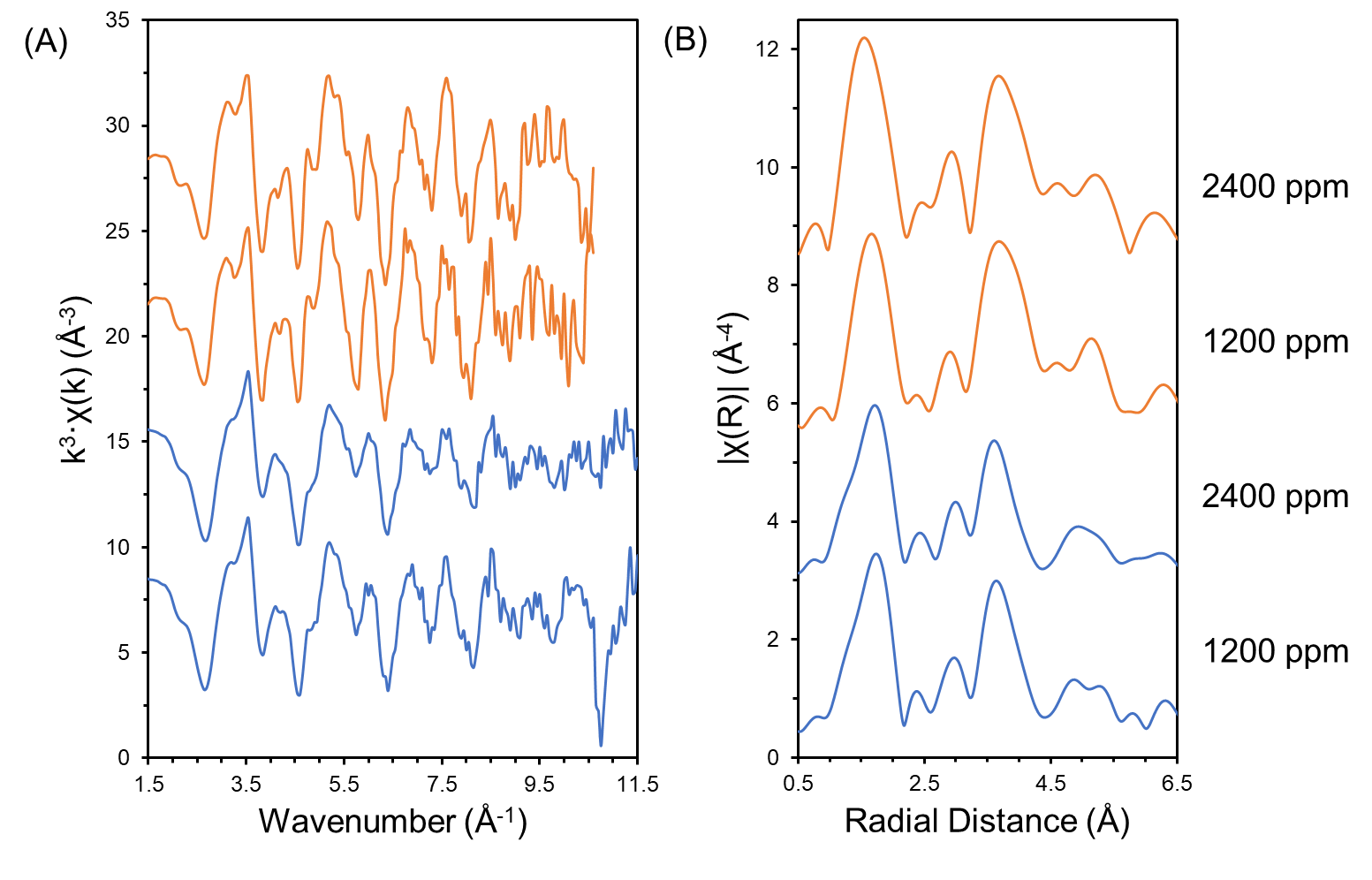


**Supplementary Figure 2. Mn K-edge XAS spectra for 1200 ppm and 2400 ppm calcined (blue) and sintered samples (orange).** (a) *k^3^*-weighted EXAFS; (b) Fourier transform of the *k^3^*-weighted EXAFS, using a Hanning window function.

**Description of EXAFS fitting of Calcined and Sintered Samples**

As discussed in the main manuscript, the fitting of the calcined samples were decided to be representative of the Mn speciation in UO_2_ for both the calcined and sintered materials. Upon comparison with the XANES (main manuscript Fig. 2) and the EXAFS (Supplementary Material Fig. 2), the environment in both sets of materials (regardless of doping concentration) are shown to be very similar.

Fitting of the EXAFS of the sintered material was attempted in this study, using both the models produced for the calcined material and alternate models (including those based upon environments determined to be present in Cr doped UO_2_) but no good fit could be achieved. The primary reason for these challenges is the low signal-to-noise ratio in the sintered samples, particularly when compared with that of the calcined material. In this sintered samples, the fitting *k*-range is limited to *k* = ~8.5 - 9 Å^-1^, whereas in the calcined material was fittable to *k* = 10 Å^-1^. These limitations on the data are manifested in the dataset due to the large amount of volatilisation that occurs during sintering and as such, whilst the nominal doping concentrations of Mn are 1200 and 2400 ppm, the actual remaining concentrations are closer to ~500 ppm (as discussed in the main manuscript).


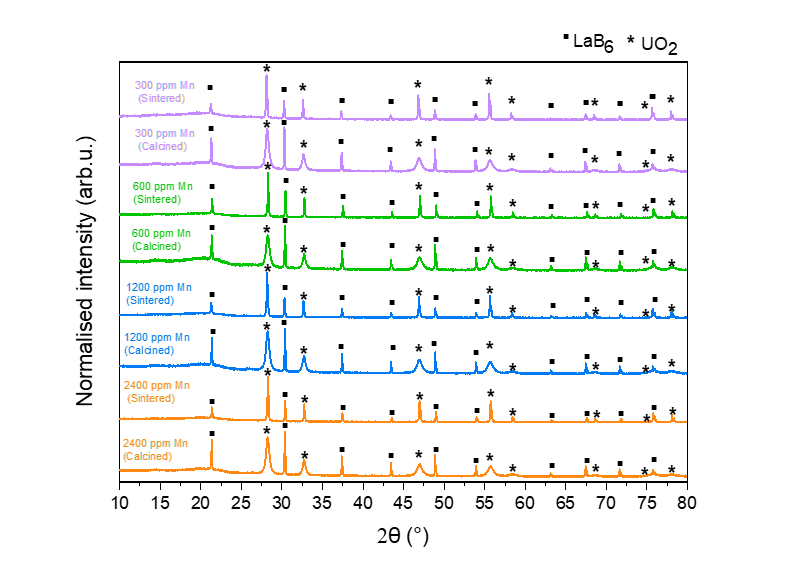


**Supplementary Figure 3. X-ray diffraction patterns of calcined and sintered material normalised using LaB_6­_ internal standard.**


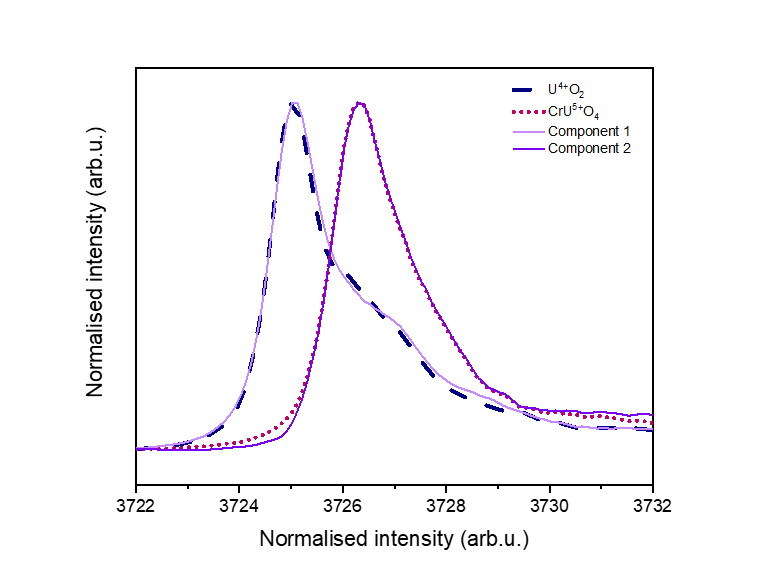


**Supplementary Figure 4. HERFD XANES U M_4_ edge spectroscopy analysis of calcined material**. Comparison between the components produced during the ITFA analysis using CrU^5+^O_4_ and U^4+^O_2_ standards.
